# Supplementary material for: Enhanced Superconducting Critical Parameters in a New High-Entropy Alloy Nb0.34Ti0.33Zr0.14Ta0.11Hf0.08
Source: Materials (Basel). 2023 Aug 24;16(17):5814. doi: 10.3390/ma16175814 (PMC10489023; doi:10.3390/ma16175814)
Supplement: Supplementary file 1 [file materials-16-05814-s001.zip › materials-2488934-supplementary.pdf]

Supplementary Materials: Enhanced superconducting critical parameters in a new high-entropy alloy $Nb_{0.34}Ti_{0.33}Zr_{0.14}Ta_{0.11}Hf_{0.08}$

Rafał Idczak [1,*], Wojciech Nowak [1,2], Bartosz Rusin [1], Rafał Topolnicki [1,3], Tomasz Ossowski [1], Michał Babij [2] and Adam Pikul [2]

1. Additional Materials and Methods

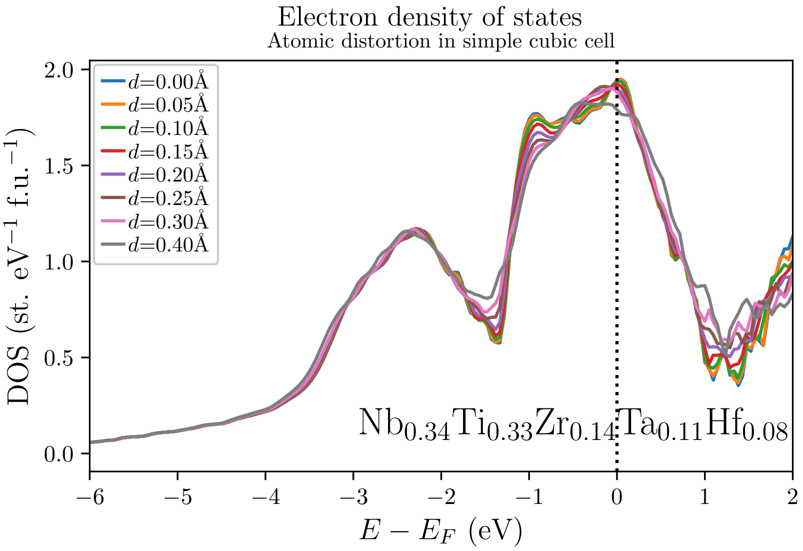

Figure S1. Impact of atomic distortion on total density of states of $Nb_{0.34}Ti_{0.33}Zr_{0.14}Ta_{0.11}Hf_{0.08}$ HEA calculated by the KKR-CPA method. The bcc structure was treated as a simple cubic lattice with two atoms basis.

To assess the robustness of the DOS to the atomic displacement we did the following experiment using the KKR-CPA framework. We treated the bcc structure of the HEA as a simple cubic lattice with two atoms basis. Next, one of those atoms was displaced for its ideal high-symmetry position in [111] direction by d, where d is ranging from 0.05 Å up to 0.40 Å. Figure S1 shows how the total DOS is changing with d. As can be seen, up to d = 0.30 Å, the DOS changes very slightly both in terms of its shape and value at the Fermi level (which is the most relevant quantity here), and only when d = 0.4 Å a marginal change of the total DOS is observed.

We ran a similar experiment, taking a simple tetragonal cell with one axis elongated twice (so to say 2 x 1 x 1 cell) containing 4 atoms (Figure S2). Each of those atoms was then displaced in all directions: the distortion in each direction was drawn from a uniform distribution on (-d, d). The conclusions are the same as previously: up to roughly d = 0.30 Å, the changes of the total DOS are minimal. It should be noticed that 0.3 Å is a very strong displacement, accounting for $\sim$10 % of the lattice parameter. It is expected that structure optimization in the DFT-PAW would not introduce such large atomic relaxations and therefore the total DOS would not be affected much. It strongly suggests, that the source of the discrepancy between theoretical and experimental DOS cannot be attributed to the atom being in ideal positions.

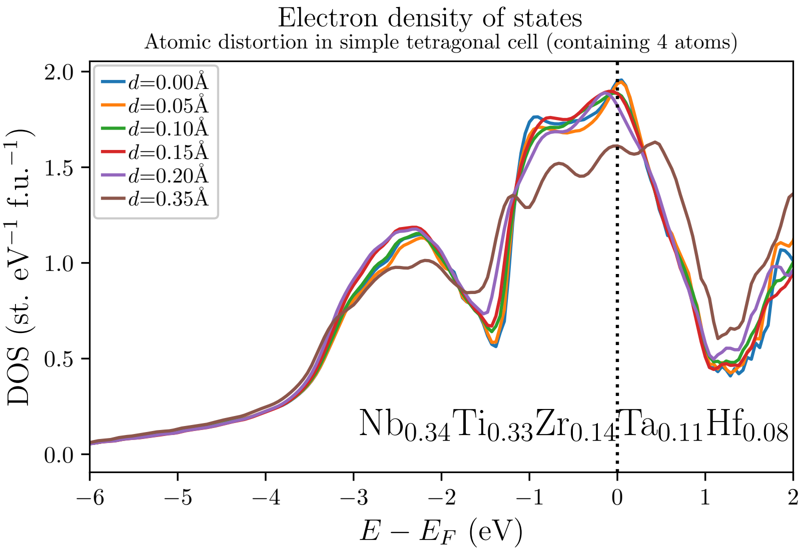

Figure S2. Impact of atomic distortion on total density of states of $Nb_{0.34}Ti_{0.33}Zr_{0.14}Ta_{0.11}Hf_{0.08}$ HEA calculated by the KKR-CPA method. The bcc structure was treated as a simple tetragonal cell with one axis elongated twice and containing 4 atoms.

2. Additional Result and Discussion

Figure S3 presents EDXS elemental mapping and determined atomic compositions for selected regions of the studied sample. The estimated uncertainty of the determined atomic compositions is close to 2 at.%.

Element	wt. %	at. %
Ti	17.91	35.02
Hf	17.16	09.01
Ta	19.77	10.23
Zr	11.88	12.19
Nb	33.28	33.54

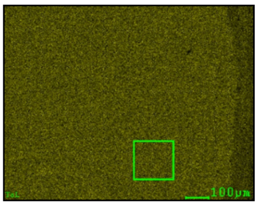

Element	wt. %	at. %
Ti	17.99	35.07
Hf	16.54	08.65
Ta	19.92	10.28
Zr	11.90	12.18
Nb	33.65	33.82

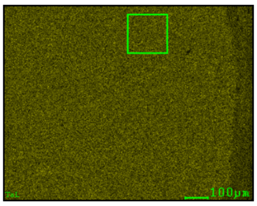

Element	wt. %	at. %
Ti	17.98	35.18
Hf	16.93	08.89
Ta	20.25	10.49
Zr	11.54	11.86
Nb	33.30	33.59

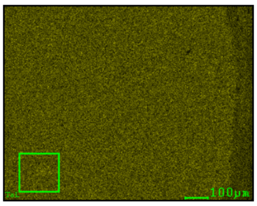

Element	wt. %	at. %
Ti	18.13	35.25
Hf	16.43	08.58
Ta	19.81	10.20
Zr	12.11	12.37
Nb	33.52	33.61

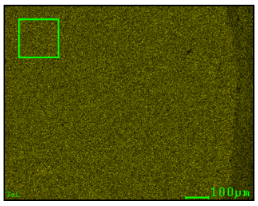

Element	wt. %	at. %
Ti	18.43	35.89
Hf	16.93	08.85
Ta	20.13	10.37
Zr	11.29	11.54
Nb	33.22	33.35

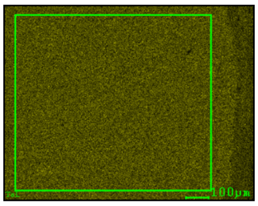

Figure S3. EDXS elemental mapping and determined atomic compositions for selected regions of the $Nb_{0.34}Ti_{0.33}Zr_{0.14}Ta_{0.11}Hf_{0.08}$ sample.
